# Liquid-Film Assisted Mechanism of Reactive Flash Sintering in Oxide Systems

**DOI:** 10.3390/ma12091494

**Published:** 2019-05-08

**Authors:** Rachman Chaim, Yaron Amouyal

**Affiliations:** Department of Materials Science and Engineering, Technion–Israel Institute of Technology, Haifa 32000, Israel; amouyal@technion.ac.il

**Keywords:** reactive flash sintering, Joule heating, oxides, liquid films, phase diagrams

## Abstract

Reactive flash sintering in oxide systems is analyzed assuming the formation of a liquid film at the particle contacts at the flash onset temperature. Formation of intermediate phases, as well as phase assemblage, are predicted upon optimal conditions of the electric field and current density. In single-phase impure oxides, the solidus and the solubility limit determine the flash onset temperature. In reacting binary systems, the composition of the liquidus determines primarily the reaction products during the cooling. In multicomponent systems, the oxide with the lowest flash temperature forms the interfacial liquid film, and the solid phase assemblage follows the equilibrium phase diagram. Examples from literature are consistent with reactive flash sintering and flash sintering assisted by a transient liquid film.

## 1. Introduction

Flash sintering (FS) is a novel technique, in which application of external electric field enables sintering and densification of ceramic powder compacts at low temperature within a few seconds. The higher the compact temperature is, the lower is the critical electric field required for the flash onset. Full densification of the powder compact often follows application of three stages of constant voltage (1st stage), flash event (2nd stage) and constant current (3rd stage). Investigation of the sintering mechanism dominating FS is of prime importance for development and standardization of this technique for more classes of ceramic materials. The two major mechanisms suggested for the ultrafast densification kinetics include solid-state sintering aided by a cascade of point defects within the bulk, i.e., Frenkel pairs [1,2,3], and liquid-film assisted sintering via the capillary forces, formed at the particle interfaces [4,5,6,7]. Two recent review papers summarized the various aspects of the FS theories [8,9]. The main claim for the solid-state approach is based on the lack of a residual amorphous phase, i.e., along the grain boundaries in the densified specimens, as well as a sudden increase in the electric conductivity at the flash temperature. However, recent long-term flash sintering experiments were successful in retaining the grain boundary and interfacial amorphous layer in monoclinic zirconia [10]. Many flash sintering studies have revealed peculiar microstructures within he grains and at the grain boundaries [11,12,13,14]. Our recent analyses showed that the formation of local liquid film at the particle contacts, due to Joule heating, is preferred energetically [15] as well as kinetically [16]. In the liquid-film assisted sintering model, the sudden increase in the electrical conductivity is associated with formation of a liquid layer, through which the electric current preferably percolates. The electric conductivity of the liquid is higher by three to four orders of magnitude compared to that of its solid counterpart near the melting point [5]. Additionally, molecular statics (MS) and molecular dynamics (MD) calculations showed negligible probability for the formation of anti-Frenkel pairs in HfO_2_ crystal subjected to electric field of 10 MV⋅cm^−1^ [17].

Reactive flash sintering (RFS) is a new approach where flash sintering simultaneously leads to the formation and densification of a new compound from a mixture of its oxide powders constituents [18,19,20]. Since the overall process involves both chemical reactions and densification, and claimed to take place in the solid-state [18,19,20], the resultant phases and phase assemblage may shed more light on the densification mechanisms, and the role of the liquid, with a correct reference to their phase diagram. In order to highlight the liquid′s role and the interfacial process character during the FS and RFS, we present and discuss two cases of binary oxide phase diagrams, for which the experimental results from the literature are available, for the sake of comparison. These include: (a) Binary phase diagram that includes one Eutectic and three Peritectic reactions similar to the Bi_2_O_3_-Fe_2_O_3_ system; (b) Binary phase diagram with negligible solid solubility, similar to the Al_2_O_3_-rich side in MgO-Al_2_O_3_ system. Additionally, we discuss the case of multicomponent oxide system subjected to RFS with respect to their phase diagrams.

## 2. Process Analysis

### 2.1. Formation of Intermediate Phase by RFS

**Figure 1:**
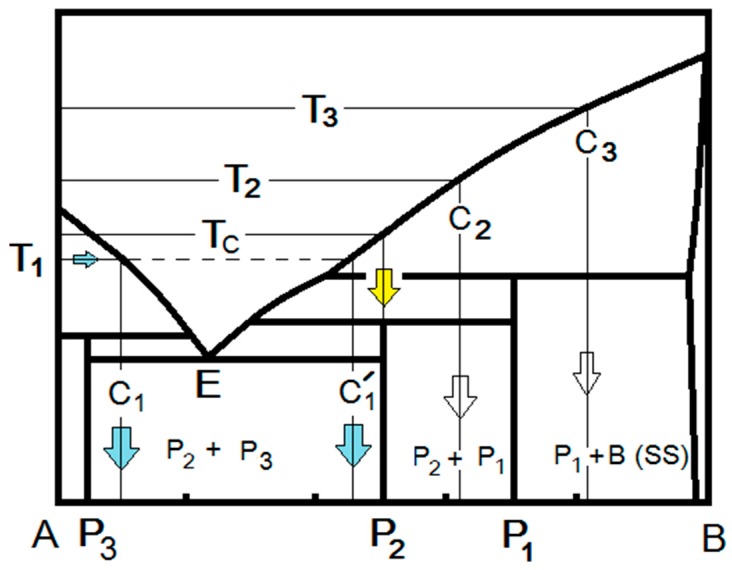
Schematic phase diagram of oxides A (Bi_2_O_3_) and B (Fe_2_O_3_). The compositions C_1_ and $C_{1}^{\prime }$ represent the phase assemblages reached at flash temperature T_1_, after short- and long-term reactive flash sintering (RFS), respectively.

Let us assume an intimate powder mixture of oxides A and B, with equimolar ratio, having a schematic equilibrium phase diagram as presented in Figure 1. To form the equimolar Peritectic phase P_2_ (AB) by RFS, the powder mixture is heated in air under constant electric field (1st stage). Based on the local-melting model at the particle contacts [5,6,7,15,16] the onset flash temperature is associated with the formation of liquid at the particle contacts (2nd stage). In our treatment, the flash temperature refers to the local temperature at the particle contacts at which liquid forms for a given electric field (and dissipated power). The local melting leads to particle wetting that, in turn, induces particle rearrangement due to the liquid-film capillary forces. We relate the rapid formation of the equilibrium phases reported during the reactive flash sintering [18,19,20,21] to immediate and full mixing of the oxide constituents within the ionic melt subjected to the high local electric field. The intrinsic tendency of the local melt that assists the local particle rearrangement is to solidify and crystallize [15,16]. Interrupted FS of pure oxides when stopping the process immediately after the flash event, i.e., 3rd stage does not exist, results in partially sintered specimens. Nevertheless, one can maintain the local melt if the local temperature is maintained at the liquidus, as is often done in the constant current regime for full densification. The temperature that develops at the melt in the interface between the particles depends on the power dissipated at the contact, which in turn depends on the applied field and the electric current at the corresponding temperature. Once the flash is ′ignited′ and the local liquid is formed, the melt composition will be defined by the liquidus at the corresponding flash temperature (i.e., C_1_ for T_1_ in Figure 1). In this regard, local melting in a binary oxide system will first take place at the contacts of the oxide particles that exhibit lower flash temperature among the two oxides (and most possibly that with the lower melting point). Dissolution of the second oxide in the local melt is immediate, and becomes intensive with time, up to the maximum solubility in liquid (i.e., $C_{1}^{\prime }$ at T_1_ in Figure 1), as determined by the liquidus at the corresponding local temperature. It is important to note that immediate and full mixing of the two oxide constituents are expected when the ionic liquid is subjected to a high electric field. Furthermore, the high applied electric-filed may induce structural order within the ionic liquid, and at the liquid-crystal interface, which in turn assists the epitaxial crystallization and growth from the melt during the cooling [16].

Another aspect of the RFS process is the time at which the specimen is maintained in the constant current regime (3rd stage). For a very short or zero durations, cooling the specimen will result in crystallization of the liquid (i.e., C_1_ in Figure 1) to the most stable solids with the phase assemblage P_2_ + P_3_ as shown by the path T_1_ → C_1_ in Figure 1, or with residual non-reacted original oxide constituent B. For long durations, the system tendency to minimize the Gibbs free energy results in dissolution of B in the interfacial liquid, according to the liquidus at the maintained flash temperature. Cooling taking place for longer durations at the 3rd stage, that are sufficient for complete dissolution of B, will result in a single phase or two-phase assemblage that correspond to the melt composition. Therefore, for longer durations at T_1_ the composition of the melt will end at $C_{1}^{\prime }$ (Figure 1) with different volume fractions of P_2_ and P_3_, compared to those resulting from C_1_. In this respect, higher flash temperatures (at lower fields), such as T_2_ and T_3_, will result in liquid compositions C_2_ and C_3_ yielding two-phase assemblages of P_2_ + P_1_ and P_1_ + B (SS) after cooling, respectively (empty arrows in Figure 1). Consequently, the formation of a single intermediate phase necessitates the refinement of the applied electric field and current to optimal conditions that correspond to a single phase and exist in the narrow (or single) composition range. Therefore, to form a pure P_2_ phase, one has to refine the flash temperature to Tc for a long duration at the 3rd stage (Figure 1).

Although the dissolution and chemical reactions are via the liquid interface subjected to high local electric field (i.e., immediate and full mixing takes place), the nanometer length scale of the powder particles shortens the durations needed for the reaction completion. One should note, that the characteristic cooling rates during FS and RFS are too slow to retain the amorphous state of the liquid at room temperature in oxides that are not glass formers [16].

### 2.2. Flash Sintering of Doped Oxide

**Figure 2:**
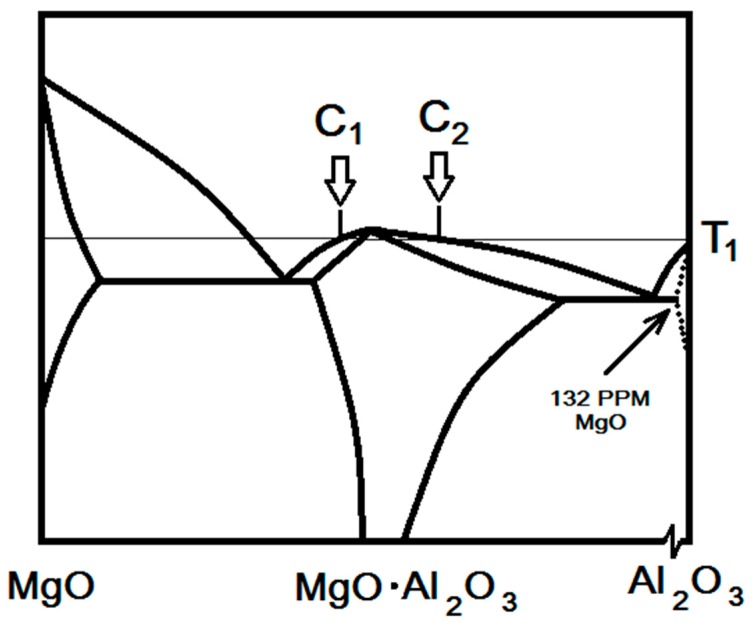
Schematic phase diagram of MgO-Al_2_O_3_. The compositions C_1_ and C_2_ show the two possible liquid compositions at T_1_ as a local temperature at the particle contacts subjected to reactive flash sintering. The Al_2_O_3_-rich side enhanced to reveal the solid solubility limit of MgO in Al_2_O_3_.

Let us assume an oxide powder with one type of oxide impurity, which indicates the limited solid solubility of the impurity in the host oxide. Negligible solubility (i.e., tens or a few hundreds of PPM in the corresponding temperature) is often considered or plotted in binary phase diagrams as zero solubility, as shown for B in A in Figure 1. In such a case, the excess impurity ions must be located at the particle surfaces (or grain boundaries when the particle is polycrystalline). Nevertheless, the true view of the phase diagram near A is as indicated (and locally magnified) by the dotted solidus and solvus lines for MgO solubility limit in Al_2_O_3_ (Figure 2), albeit at extremely low concentrations. Therefore, once liquid is formed at the particle contacts due to local Joule heating, the composition of the interfacial melt is defined by the liquidus at the corresponding flash temperature. This temperature is usually lower than the melting point of the pure oxide, since the liquidus line denoting the melting temperature usually decreases with the increase of solute concentration. Consequently, oxides with impurities can be flash sintered at a lower flash temperature or applied electric fields. We note that this behavior depends on the thermodynamic interaction parameters between the atomic species constituting both oxides as well as the liquid phase. The tendency to form solution from pure oxides is determined by the balance between entropy and enthalpy of mixing. As the former always encourages mixing, the latter may either encourage or discourage mixing, depending on the interaction parameters, which can be either negative (attractive) or positive (repulsive), respectively [22]. For the first case, the negative enthalpy of mixing results in decrease of the melting temperature upon addition of solute elements. Conversely, in the second case the enthalpy of mixing is positive, which results in increase of the melting temperature with additions of solute elements. When the conditions of the first case are fulfilled, the solute concentration in the liquid phase is greater than that of the solid phase, meaning that solute transport via the melt is activated, which enables liquid phase sintering. These conditions were thoroughly discussed by German et al. [23]. The resultant grain size in the densified specimen depends on the original particle size, as well as the duration at the 3rd stage of the flash sintering.

### 2.3. RFS in Multicomponent Systems

The immediate outcome of the previous sections is that the oxide component with the lowest flash temperature is the first to melt locally in the multicomponent system. Therefore, the phase evolution and the final phase assemblage in such systems depends on the chemical reactions that exist in the multicomponent phase diagram. In principle, the formed liquid film assists the chemical reactions to the maximum solubility at the corresponding flash temperature and the following cooling results in solidification to the most stable solid at room temperature. In the following section, we will discuss this point further, based on examples from literature.

## 3. Discussion

In this section, we will examine the liquid-film assisted model for RFS and FS by observation of the experimental phase evolution for different systems reported in the literature. Perez-Maqueda [18] and Gil-Gonzalez [20], from the same groups, performed comprehensive investigations of RFS towards pure BiFeO_3_. Equimolar compositions of Bi_2_O_3_ and Fe_2_O_3_ powders, of either coarse micrometer-size mixtures [20] or high-energy ball milled nanopowders [18], were reactive flash sintered under electric fields of 50 to 150 V⋅cm^−1^, at different current densities of 20 to 50 mA⋅mm^−2^ [18,20]. The ball-milled powders exhibited flash temperatures of 420, 500, and 600 °C for the electric fields of 150, 100, and 50 V⋅cm^−1^, respectively [18]. Nevertheless, as was discussed in many previous studies, the local temperature at the particle contact, as well as the average temperature of the radiating specimen, may be much higher than the furnace temperature, from which the flash temperature is determined. In this respect, calculation of the specimen temperature from the power peak, assuming black body radiation (BBR), resulted in 941, 860, and 897 °C for the fields of 150, 100 and 50 V⋅cm^−1^, respectively. The calculated temperatures were higher by 300 to 500 °C than the corresponding flash temperatures. The Bi_2_O_3_-Fe_2_O_3_ phase diagram [24] essentially is similar to Figure 1, where P_1_ is Bi_2_Fe_4_O_9_, P_2_ is BiFeO_3_ and P_3_ is Bi_25_FeO_39_ and the above calculated temperatures (941, 860, and 897 °C) are located above the Peritectic temperatures of 937 °C for P_1_ (i.e., L + B (ss) → Bi_2_Fe_4_O_9_) and 852 °C for P_2_ (i.e., L + P_1_ → BiFeO_3_) respectively. Therefore, when the particle contacts melt, one can maintain the local melt for longer durations at these calculated temperatures. The authors found a multi-phase assemblage of BiFeO_3_ + Bi_25_FeO_39_ + Bi_2_Fe_4_O_9_ at fields below 15 V⋅cm^−1^, and single pure BiFeO_3_ at higher fields [18].

In their previous research of ball-milled nanopowders, Perez-Maqueda and coworkers [20] found optimal RFS parameters of 50 V⋅cm^−1^ and 35 mA⋅mm^−2^ at 625 °C, which yielded single phase BiFeO_3_ (i.e., P_2_ phase in Figure 1). Other combinations of the electric field and current (i.e., different dissipated power, hence different liquidus temperatures) led to different phase assemblage. In this respect, the following phase assemblages were observed with increasing the current at 50 V⋅cm^−1^: BiFeO_3_ + Bi_25_FeO_39_ (i.e., P_2_ + P_3_ in Figure 1), single-phase BiFeO_3_ (P_2_ in Figure 1), and less BiFeO_3_ + more Bi_25_FeO_39_ + Bi_2_Fe_4_O_9_ (i.e., P_2_ + P_3_ + P_1_) [20]. This phase evolution is in full agreement with our analysis in Section 2.1, assuming that the actual liquidus temperatures, reached at each of these different current densities, are between 780 °C–852 °C, 852 °C–937 °C, and above 937 °C, respectively.

Moreover, increasing the field at constant current density (35 mA⋅mm^−2^) from 50 to 100 and 150 V⋅cm^−1^ [20] resulted in single phase BiFeO_3_ (P_2_) at the lower field, followed by BiFeO_3_ + Bi_25_FeO_39_ (P_2_ + P_3_) phase assemblage at higher fields (i.e., lower flash temperatures). These fields were associated with flash (furnace) temperatures of 625, 595, and 578 °C, respectively. According to our analysis, the local liquid temperature is around 1020 °C (exactly above the P_2_ composition), for the flash temperature of 625 °C. The other two flash temperatures are higher, as well, and should be located along the liquidus line in the 937 °C–852 °C and 852 °C–780 °C ranges, respectively. In view of the 300 °C to 500 °C difference found between the measured and the calculated temperatures, these phase evolutions follow our expectations in accordance with the liquid-film concept.

Interrupted RFS experiments versus temperature confirmed the formation of Bi_25_FeO_39_ (P_3_) from a reaction of Bi_2_O_3_ with Fe_2_O_3_ together with a residual Fe_2_O_3_ up to 470 °C [20]. Further temperature increase resulted in formation of BiFeO_3_ (P_2_) and dissolution of Fe_2_O_3_. The authors also reported that some liquid formed at the coarse particle surfaces [20]. We assume that the local particle contact temperatures under the field are higher than the furnace temperature; hence, the observed phase evolution is consistent with the formation of a liquid-film that assists the chemical reactions.

Another indication of the liquid-film assisted RFS is the formation of spinel (MgAl_2_O_4_) reported by Kok et al. [21]. They reported the formation of two different compositions of spinel, MgO⋅1.5Al_2_O_3_ and MgO⋅3Al_2_O_3_, in the same specimen subjected to RFS. Increasing the electric current (and dissipated power) for shorter durations led to the evolution of a single composition spinel. Solid-state reaction between MgO and Al_2_O_3_ should lead to spinel with a single composition at the interface. Therefore, the observed phase evolution cannot be explained by solid-state reaction/sintering. Nevertheless, the formation of two spinel compositions is straightforward in the presence of local liquid-film at the particle contacts, the temperature of which is above the congruent temperature of spinel, 2135 °C [25] (Figure 2). As mentioned above, immediately after the local melting, the local contact temperature defines the local composition according to the liquidus line. In the case of spinel formation, any decrease in the local temperature (i.e., down to T_1_ in Figure 2) moves the melt composition along the two liquidus lines (compositions C_1_ and C_2_ in Figure 2), away from the congruent temperature (2135 °C). This, in turn, results in the formation of two spinel phases during the cooling, the compositions of which are determined by the two liquidus lines at the flash temperature. The higher current density for shorter durations is expected to increase the dissipated power, hence to increase the local contact temperature. Consequently, if the local temperature increases above 2135 °C for short durations, the more stable spinel (with lower solubility) will be formed during cooling, i.e., the spinel with lower eutectic temperature adjacent to its congruent temperature. The Al_2_O_3_-spinel eutectic temperature (~1925 °C) is lower than the eutectic MgO-spinel temperature (~1995 °C) [26,27], hence Al_2_O_3_-rich spinel (i.e., MgO⋅2.5 Al_2_O_3_) is expected, in agreement with the experimental findings [21].

The last example in this category of RFS is the formation of Al_2_TiO_5_ from Al_2_O_3_ and TiO_2_ binary powder mixtures subjected to reactive flash sintering at 900 °C and 500 V⋅cm^−1^ [19]. However, assuming black body radiation, they calculated the specimen temperatures as 1150 °C and 1310 °C for current densities of 20 and 25 mA⋅mm^−2^, respectively. Using X-ray diffraction, the authors related the formation of the Aluminum titanate to the current-controlled stage (3rd stage). Nevertheless, careful observation of the presented SEM (scanning electron microscope) image (Figure 7 in ref. [19]) reveals irregularly curved grain boundaries, with a few smaller grains containing typical eutectic microstructure. Similar to magnesium-aluminate spinel, Al_2_TiO_5_ in the Al_2_O_3_-TiO_2_ phase diagram also solidifies in a congruent manner, however to a very stoichiometric composition in contrast to the wide composition rage of spinel (MgAl_2_O_4_). According to our analysis of the liquid-film assisted RFS, the reaction between Al_2_O_3_ and TiO_2_ in the equimolar powder mixture could yield single pure Al_2_TiO_5_ phase, provided the reactive flash sintered conditions are strictly controlled. Consequently, in most cases RFS should yield two-phase assemblage of Al_2_TiO_5_ with eutectic microstructure of TiO_2_ + Al_2_TiO_5_, since this eutectic reveals the lower eutectic temperature (~1700 °C) among the two eutectic reactions that exist in this system [28]. To our opinion, the observed irregularly curved grain boundaries (Figure 7 in ref. [19]) are the remnants of the liquid-film assisted RFS mechanism. The grains with eutectic microstructure are the evidence for the presence of the liquid during the process.

Next, we will discuss the rapid densification of an oxide with impurity since in most cases the impurity reduces the flash sintering temperature compared to that of the pure oxide. Alumina is a good candidate, since its flash sintering was investigated both as a pure oxide and after doping with MgO [1,29]. Different investigators determined the solubility limit of MgO in Al_2_O_3_ single crystals and polycrystals by different techniques [30,31,32,33]. Miller et al. [33] determined this solubility limit to be 132 ± 11 ppm at 1600 °C (i.e., assigned in Figure 2) using quenching experiments and local wavelength dispersive spectroscopy (WDS) analyses of the bulk and the grain boundary compositions in SEM. Their analyses indicate excess of 2 to 3 Mg ions per nm^2^ at the grain boundaries. The corresponding MgO concentration in the MgAl_2_O_4_ spinel, which, in turn precipitates at the grain boundaries due to excess MgO is between~10 to 17 wt.% at 1600 °C [33]. Therefore, most alumina powders with 0.25 to 0.5 wt.% MgO sintering additive [1,29] should contain excess MgO at their particle surfaces if single crystal, or along their grain boundaries if polycrystalline. These MgO-rich surfaces and interfaces exhibit lower melting points than pure Al_2_O_3_, following the solidus line of Al_2_O_3_ with liquid (dotted line in Figure 2). Therefore, when the particle contact melts due to the local Joule heating, the melt dissolves the excess MgO at the particle surface; hence, one can maintain the liquid (at 3rd stage) at much lower temperatures (i.e., along the liquidus of alumina solid solution + liquid in Figure 2) than the pure Al_2_O_3_ melting point. Indeed, 0.25 wt.% MgO-doped alumina samples were flash sintered at temperatures of 1260 °C and 1320 °C, using electric fields of 1000 and 500 V⋅cm^−1^, respectively, whereas no FS took place in pure Al_2_O_3_ [1]. Nevertheless, arcing and sparks were emitted from nanopowder compact of pure Al_2_O_3_ flash sintered at 1400 °C and 1000 V⋅cm^−1^ [1], which is interpreted as evidence for the local dielectric breakdown [34], hence melting. This ceramic behavior is consistent with Mishra et al. [35] deduction that the dielectric properties are critical for successful densification of TiO_2_-doped alumina by plasma activated sintering. Flash sintering of 99.8 wt.% pure alumina containing 0.115 wt.% of MgO, Na_2_O, Fe_2_O_3_, SiO_2_, CaO and other impurities, exhibited lower flash temperature of 1000 °C at 1000 V⋅cm^−1^ using platinum electrodes [34]. This further indicates the importance of the impurities in lowering the local melting temperatures of the particle contacts, where the excess solute ions are concentrated.

While the lack of a residual amorphous film at the grain boundaries after FS is due to the epitaxial solidification in pure oxide systems [16], one may observe a residual glass when the doping elements are glass formers. In this respect, FS of 90 wt.% pure alumina powders containing 8 wt.% SiO_2_ (glass former) and 2 wt.% MgO at 1000 V⋅cm^−1^ revealed flash temperature of 1090 °C [36]. More important was the formation of MgO-rich region, near the cathode, where the Mg/Al composition ratio was close to 1, consistent with local melting and Mg enrichment. Our analysis implies that the electric field and the flash do not alter the thermodynamics of the system, albeit they extremely promote the reaction and densification kinetics towards equilibrium, assisted by the liquid film. In this respect, the electric field acts as another source of energy, similarly to temperature and pressure.

Finally, our analyses show that for a general case of multicomponent oxide system, the oxide component with the lowest flash temperature is expected to melt first. As such, the expected chemical reactions in the multicomponent oxide system subjected to RFS are dictated by the corresponding liquidus temperatures between the melting oxide and the rest of the components in the multicomponent phase diagram. Recently, Yoon et al. [37] used pure 0.2 μm Al_2_O_3_ particles with 1 μm MgO powders mixed with 50 vol.% of nanometric 8YSZ (ZrO_2_ stabilized with 8 mol.% Y_2_O_3_) to form spinel (MgAl_2_O_4_) by reactive flash sintering at 960 °C at increasing current mode [38]. They recorded the densification and the phase transformation using in-situ X-ray diffraction. Although 8YSZ was used to initiate (′ignite′) the flash, no peaks from this oxide were recorded during the course of the experiment where phase transformation preceded the sintering [37]. One should note that chemical reactions are associated with activation energies of a few hundreds of KJ⋅mol^−1^ compared to a few KJ⋅mol^−1^ involved in sintering. Following the fundamental properties of 8YSZ, MgO and Al_2_O_3_, such as crystal-type, cationic potential and fusion entropy, their comparative flash temperatures may be ranked as MgO > Al_2_O_3_ > 8YSZ [39]. The same fundamental properties also determine the phase relationships within the equilibrium phase diagrams through the Goldschmidt ′structure-fields maps′. Following the ternary ZrO_2_-MgO-Al_2_O_3_ equilibrium phase diagram [40] and our present approach, one expects for zirconia-melt mediated chemical reactions, accelerated by the local applied field (i.e., via liquid–solid electro-wetting and electro-mixing in the liquid). The resultant expected phase is spinel with high Zr ions content, provided full solubility reached during the chemical reaction.

## 4. Conclusions

In conclusion, the aforementioned case studies documented in the literature can be elucidated in terms of presence of a liquid-film during reactive flash sintering. Formation of an intermediate phase or phase assemblage at certain composition by reaction flash sintering necessitates homogeneous heating of the powder compact to achieve the liquidus of that composition.

## Abbreviations

The following abbreviations used in this manuscript:

BBR	Black body radiation
FS	Flash sintering
RFS	Reaction flash sintering
SEM	Scanning electron microscopy
WDS	(X-ray) Wavelength dispersive spectroscopy
